# Integrating Systems Thinking in Nutrition and Dietetics Education

**DOI:** 10.1111/jhn.70036

**Published:** 2025-03-10

**Authors:** Erin Bergquist, Lyndi Buyckingham‐Schutt, Scott Smalley, Christina Gayer Campbell, Awoke Dollisso, Shuyang Qu

**Affiliations:** ^1^ Iowa State University Ames Iowa USA

**Keywords:** dietetics curricula, nutrition and dietetics education, sustainable food systems, systems thinking

## Abstract

**Introduction:**

Integrating systems thinking, which emphasizes recognizing interconnections, gaining diverse perspectives and considering the big picture, can enhance curricula and better prepare practitioners. Despite its benefit and growing support, systems thinking is not yet a required part of dietetics accreditation or entry‐level practice. This qualitative study sought to understand educators' views on incorporating systems thinking into nutrition and dietetics education.

**Methods:**

Thirteen Registered Dietitian Nutritionists from various US census regions and accredited program types were recruited. Semi‐structured interviews were audio‐recorded and transcribed verbatim. Grounded theory methodology and iterative coding analysis were used to analyse the transcriptions in Excel.

**Results:**

Three interrelated themes emerged from educator perspectives: (1) individual characteristics (personal attributes; multi‐, inter‐ and transdisciplinary experiences; perspective seeking; relationship building), (2) nutrition and dietetics education (training, resources, accreditation requirements, value awareness) and (3) the dietetics profession (organizational culture, education and practice integration, continuing education, professional guidance).

**Conclusions:**

Integrating systems thinking into nutrition and dietetics education can be facilitated across three levels: individual, education and the profession through training, resources, accreditation competencies and awareness. However, implementation requires fostering a cultural shift within the profession and overcoming resistance to change.

## Summary

- A qualitative study with insights into US educators' perspectives of integrating systems thinking into nutrition and dietetics education.
- US educators who have integrated systems thinking experienced challenges of inadequate resources, professional development, time and competency support.
- Our findings emphasize the importance of a systemic approach between individual, educational and professional levels to prepare future dietitians for complex challenges.

## 1 Introduction

In nutrition and dietetics, it is essential to empower future professionals with the necessary skills to tackle real‐world challenges that support the Sustainable Development Goals and involve systemic changes across agriculture, nutrition and healthcare sectors [1]. Reaching these goals necessitates an advanced and cross‐disciplinary skillset and curricula addressing broader societal, environmental and economic contexts influencing food environments. Dietitians, as nutrition experts, must also navigate the complexity that extends beyond food environments and into systems such as healthcare systems, where addressing complex challenges can improve the quality of care [2, 3]. However, a recent study of dietetics graduates revealed limitations in their training, highlighting a focus on clinical dietetics, leaving them unprepared for diverse areas of practice [4]. To prepare future practitioners to face complexity across sectors and within complex systems, integrating systems thinking, a problem‐solving approach marked by an emphasis on big‐picture thinking, diverse perspective seeking and recognizing interconnections within systems elements [5, 6, 7, 8, 9, 10, 11], offers promise.

Systems thinking is both a skillset and pedagogical framework that improves the ability to recognize complex systems behaviour to improve intervention and outcome strategies [6, 9, 12, 13]. Its tenets include a holistic perspective of a dynamic system, seeking patterns within the system, transformative insight, self‐examination and action [7, 14]. Educators in nutrition and dietetics have described systems thinking using terms and skills such as a holistic approach to complexity, obtaining diverse perspectives and examining interrelationships, which build upon strong pedagogical foundations [15].

Systems thinking is considered an essential curricular component in sustainability education [16, 17, 18]. Many disciplines, including agriculture [19, 20, 21]; business [22]; healthcare and medicine [23, 24, 25, 26, 27]; policy [28]; public health [29]; science, technology, engineering and mathematics (STEM) related fields [30, 31, 32, 33]; and STEM education [34, 35, 36] have integrated it into their curriculum. Systems thinking approaches are both student‐centered and beneficial to instructors because they facilitate understanding complexity [37, 38], problem‐solving [7, 24, 39], student engagement [40] and assist with the collaborative learning process [41]. Despite these benefits, educators have faced various challenges with integrating systems thinking into higher education curricula [15, 42], including managing complexity [38].

Interest in systems thinking is emerging in the dietetics field within areas such as sustainable food systems education [11, 43, 44, 45], healthcare systems [2] and public health [46]. A recent qualitative study found that dietetic educators who teach systems thinking in accredited nutrition and dietetics programs experienced a range of benefits, including improved skill sets in navigating complexity, leadership opportunities, connecting to personal values and establishing an equity mindset [15]. In addition, research has shown that while few accredited program directors teach it in their courses, the majority support including it in curricula [43].

Although integrating systems thinking into nutrition and dietetics is gaining support, it is not included in accreditation or entry‐level practice requirements in the United States (US) [47, 48, 49, 50, 51, 52] or across the globe [53, 54, 55]. This research study aimed to explore educators' perspectives on integrating systems thinking in nutrition and dietetics education.

## 2 Materials and Methods

This study was part of a research project that explored the experiences of Registered Dietitian Nutritionist (RDN) educators in the US when teaching systems thinking in accredited nutrition and dietetics programs. Previous findings highlighted how educators used systems thinking to teach in nutrition and dietetics and the benefits of its integration into education [15]. This study focused on educators' perspectives on systems thinking integration into curricula.

The following research questions guided this study:

1. What could facilitate or empower RDN educators to use systems thinking in nutrition and dietetics education?
2. What are the gaps regarding dietetic educators integrating systems thinking in nutrition and dietetics education?

The research team included a primary researcher, a doctoral candidate in Agricultural Education with 21 years of experience as an RDN and 13 years of nutrition and dietetics teaching experience (E.B.). Additionally, the primary researcher developed practice and educational documents about sustainable food and water systems, as well as systems thinking in nutrition and dietetics. The research team also included a PhD, RDN, whose experience includes research, teaching in higher education and serving as a dietetic preceptor (L.B.‐S.), and a PhD researcher specializing in experiential learning, teacher preparation and professional development (S.S.).

Ethical approval was obtained from the Iowa State University Office of Research Ethics, Institutional Review Board (ID #23‐257). The study quality was appraised using the Consolidated Criteria for Reporting Qualitative Research (COREQ) checklist [56].

The researchers adopted a constructivist theoretical perspective, understanding that people interpret situations differently based on their experiences and perspectives [57]. The primary researcher created a semi‐structured interview guide that included questions about facilitators and gaps in integrating systems thinking in nutrition and dietetics education [58]. To ensure the guide's content validity and reliability, it was pilot tested with two RDNs who had experience teaching systems thinking in the US in Accreditation Council for Education in Nutrition and Dietetics (ACEND) accredited Didactic Programs in Dietetics. Following pilot testing, minor changes were made to the guide, including omitting one question for redundancy and revising two questions to improve clarity.

### 2.1 Participants, Recruitment and Sampling

RDNs teaching systems thinking in an ACEND program in the US were eligible for inclusion in this study. The sampling strategy involved a combination of purposive and theoretical sampling techniques. Purposive sampling identified 10 educators who had worked with the US Academy of Nutrition and Dietetics Foundation's sustainability initiatives, prioritizing participants to represent each accredited program type (Coordinated, Didactic, Graduate, Dietetic Internship) and US census tract geographic region (Midwest, Northeast, South, West). During this process, one participant declined an interview, one did not respond to a request, and one experienced rescheduling difficulty. Theoretical sampling was then carried out using a snowball sampling technique, where the researchers relied on the expertise of initial participants to identify other educators [59]. Data collection and analysis were carried out simultaneously to identify emerging experiences or themes [60]. After conducting 11 interviews, theoretical saturation was achieved as no new information surfaced. Two additional interviews were conducted to ensure saturation for a total of 13 participants, which was a consensus decision by the research team.

### 2.2 Procedures

Once initial study participants were identified, they were solicited via email to participate in the study. The invitation email described the study and provided informed consent. Interested participants responded to the email, and a follow‐up email provided interview times. Participants provided consent by scheduling an online interview. Interviews were conducted using Zoom Video Communications Inc. (San Jose, CA). Immediately before the interview, participants completed an online demographic survey, collecting information such as gender, years of experience as a dietitian, years teaching in an accredited program, years teaching systems thinking, race and ethnicity. The primary researcher (E.B.), a PhD candidate with qualitative research experience, completed interviews between September and November 2023. The researcher described the purpose of the study to the participants before interviewing them using the semi‐structured guide. Interviews were recorded and transcribed by an external transcription service to enhance dependability (Otter.ai Inc., Mountain View, CA). The primary researcher ensured the accuracy and descriptive validity of the data by meticulously reviewing and comparing the transcriptions against the audio recordings, rectifying any omissions, and incorporating notes taken by hand. To maintain confidentiality, transcripts were deidentified before being uploaded to a multi‐factor authenticated cloud storage system (Box Inc., Redwood City, CA) and manually coded using an Excel worksheet (Microsoft Corp., Redmond, WA).

### 2.3 Data Analysis

Data were analysed using grounded theory methodology in three steps: initial coding, focused coding and theoretical coding [60, 61]. After consulting with the research team regarding coding on the first three transcripts, the primary researcher examined the first seven transcripts and developed the initial coding list. After discussing and revising the initial coding list with the research team, the primary researcher continued the process of interviewing, carefully transcribing data and adding to the initial coding list. The list was then synthesized through focused coding to develop categories while still considering the initial codes. The primary researcher created analytic memos as a fundamental tool in the analysis process, which underpins the classic grounded theory approach to theme development [60], enhancing reflexivity and confirmability [62]. Throughout the analysis, the research team held regular meetings (E.B. and S.S. and E.B. and L.B.‐S.) and employed triangulation techniques to increase validity [63]. Through discussion and consideration of contradictory findings [64], the research team conducted theoretical coding to categorize the interrelationships among themes [61]. The primary researcher then elaborated on these initial themes by incorporating relevant participant quotes. Subsequent discussions among the research team focused on refining theory, with particular attention to the relationships between the themes. This iterative process continued until a consensus was reached on the final themes [61].

## 3 Results

This study explored the perceptions of RDN educators about integrating systems thinking in nutrition and dietetics education (Table 1). Interviews averaged approximately 52 min and educators represented each accredited program type and geographic region in the four US census tracts. All participants (*n* = 13) were White, and the majority were women (*n* = 12). They averaged 23 years of experience working as an RDN and 13 years of experience teaching in an accredited program. While all educators reported teaching systems thinking, nearly half of educators (46%) reported teaching it for more than 6 years.

**Table 1:** Characteristics of registered dietitian nutritionist educators who participated in semi‐structured interviews about teaching systems thinking in the US (*n* = 13).

Characteristic	*n* (%) or mean ± SD
**Accredited program type**	
Coordinated program (CP)	4 (31%)
Didactic program (DPD)	2 (15%)
Dietetic internship (DI)	4 (31%)
Graduate program (GP)	3 (23%)
**Institution type**	
College or university	12 (92%)
Academic Medical Center	1 (8%)
**Geographic location (US region)** a	
Midwest	3 (23%)
Northeast	3 (23%)
South	2 (15%)
West	5 (38%)
**Years teaching systems thinking**	
< 1 year	2 (15%)
1–5 years	5 (38%)
6–10 years	3 (23%)
11–15 years	2 (15%)
16–20 years	1 (8%)
**Years as a credentialed RD/RDN**	23.6 ± 11.1
**Years teaching in accredited program**	13.5 ± 8.5
**Gender identity**	
Woman	12 (92%)
Man	1 (8%)
**Race and ethnicity** b	
White or Caucasian	13 (100%)
Other	1 (8%)

^a^ US regions defined in accordance with the US Census Bureau, Census Regions and Divisions of the US [65].

^b^ Participants were allowed to ‘select all that apply’, ‘other’ or ‘prefer not to answer’. This table condensed selected options to ‘other’ to protect participant privacy.

**Figure 1:**
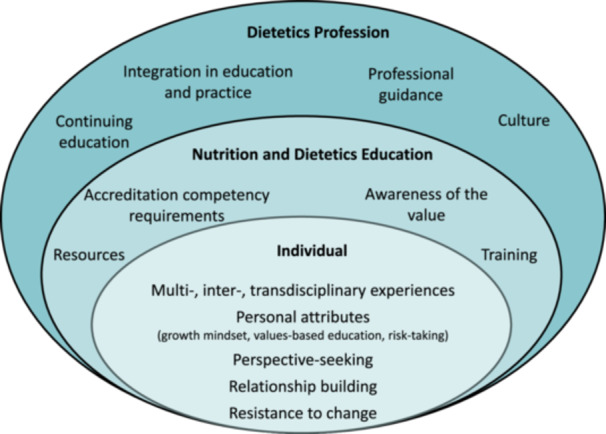
A theoretical framework depicting nutrition and dietetic educator perspectives about systems thinking integration across three interrelated themes: (1) individual, (2) nutrition and dietetics education, (3) dietetics profession (*n* = 13).

Educator perspectives on integrating systems thinking in nutrition and dietetics education emerged across three themes [1]: individual [2], nutrition and dietetics education and [3] dietetics profession. A theoretical framework highlights the interconnectedness between these themes, suggesting that changes at any level can influence the others (Figure 1). This interconnectedness reflects principles similar to those of the socioecological model, underscoring the complexity and bidirectional nature of nutrition and dietetics education and practice. Quotes for each theme are included in the text, with additional illustrative quotes provided in Table 2.

**Table 2:** Registered dietitian nutritionist educators perspectives about integrating systems thinking in accredited nutrition and dietetics programs (*n* = 13).

Theme, category and subcategory	Representative quote
**Individual Characteristics**	
Personal attributes:	
Growth mindset	‘It was very clear when I was doing on‐the‐ground work, and when I was doing the policy work, like this is a band‐aid, and it was frustrating work. And I guess that's what pushed me to think more about the system’. P1
Values‐based education	‘One thing that I posed to [students] was, “In a time of crisis, a lot of people focus on children, but a lot of cultures need to focus on adults. So, what do you think about that?” It challenges worldviews’. P7
Risk‐taking	‘I think what has really helped me is to not be afraid to try… to constantly reevaluate and use that iterative process to make improvements along the way. I'm into figuring out what you want, or what you're passionate about, and having the confidence to make a change’. P5
Multi‐, inter‐, transdisciplinary experiences	‘I ended up working with this program [for k‐12 children]. I was doing all these field trips and all these experiences with kids where we were going to farmers' markets and cooking and composting all of the leftovers and talking about food processing and talking about all the natural resources that are used to make food packaging and so it was very concrete and hands‐on for children’. P8 ‘Working with farmers and communities and working with low income and working overseas and seeing how food is produced and preserved…interaction with people in the food system, across all parts of the food system, but particularly consumers and producers, has really helped me, personally, to understand the importance of systems thinking’. P6
Perspective‐seeking	‘To be a better dietitian, you need to understand the outside things are influencing people's food choices and nutritional health choices’. P7
Relationship building	‘I think involvement in [dietetics practice groups] that naturally think this way, and have been leaders for decades, trying to push…others to be thinking in this way. Just the person‐to‐person way, and even in this conversation, it's like, “oh, here I can be more intentional” in how I think and design this’. P11
Resistance to change	‘Yeah…as a professor, we are most comfortable teaching how we learned. And so I think that anyone who did not learn this and like I said, I definitely didn't learn any of it in my undergraduate or master's degree at all. I think it would be really uncomfortable to have to take on something that seems outside and isn't something that you know about – it's very hard’. P8
**Nutrition and Dietetics Education**	
Training:	
Multi‐, inter‐, transdisciplinary training	‘My training is mostly systems approach. My undergrad is very systems oriented in wildlife biology. One of my masters is in horticulture and then thinking about like the broader ecological agroecology system, so that was already embedded. And I was fortunate with my doctorate to be able to take classes in the school of sustainability, and I was maybe the first in my cohort to do that…so I think I entered dietetics already from a systems thinking perspective’. P2
Adult learning methods	‘Understanding how to educate people…formal training in adult learning methods, designing experiences and giving feedback’. P6
Sustainable food systems training	‘[My master's degree is in] sustainable food systems’. P13
	‘One challenge is just the lack of knowledge [in sustainable food systems]. It's a big base of knowledge that you need to have to at least access things, and it's a lot to ask when our curriculum is already full’. P6
Lack of systems thinking training	‘I imagine that if I were trying to implement [systems thinking] for the first time, I probably would be struggling with it. “Where do I even begin and what are the resources? How do I know what's right or not?” There's not a well‐established path or metrics to say ‐ now I'm a systems thinker’. P4
Limited educator skillsets in teaching how to navigate complexity	‘People don't know how to circumvent [complexity], and or they don't have the confidence to say this is something we could change. So, what does that require? It requires knowledge, it requires data, it requires coming up with a plan, it requires implementing the plan on a pilot basis, looking at whether or not you are able to do it ‐ was it effective, whether it's feasible, going back and negotiating with every prospective change and then trying to scale things up. So, I think the barriers to systems thinking are ‐ sometimes the problems seem overwhelming, you get that deer in the headlights paralyzed state. This is such a big problem. We can't do anything about it’. P5
Resources:	
Curricular resources	‘I could see some kind of a book could even accompanied with case studies… I think a faculty member could use this in their talks’. P12
	‘I also think that if there were some translational materials to show us how the ADIME model can be systems thinking, right? It's a model, but it's not it's not quite systems thinking unless you make it that’. P13
Perceived adequate time	‘I think people need the time. I think people need the time to be contemplative. And I think so many of us are so overextended that it's hard to sit back and just think about what needs to be done, and we need to be proactive versus reactive to situations. I think as a clinician and as educators of our future dietetic professionals, we've got a lot in front of us that is challenging’. P5
	‘Change is hard…we'll see things all the time, “I should add that to my class, I should change my class,” but you know the environment right now, it's awfully hard to do that when you've got various [student learning outcomes] and measures. When you're trying to mirror a campus in an online course, to say, “Oh, let's interject this new framework.” It makes sense, but the time and effort to do it is an obvious challenge’. P9
Mentorship/support	‘I think having colleagues who also are thinking about systems that has been absolutely wonderful. If we could figure out some kind of support group, with stages, “you're at the beginning stages here. You're an intermediate, and here you are more advanced.” I think that it can't just be educators… [we shouldn't be] separating education from community and what's happening within the community’. P3
	‘Having mentors or others in the field who are already doing that work as examples, there's a way that people can realize what it is orthodox’. P12
Lack of professional development funding	‘I'm surprised and shocked at how little time or recognition is given to professional development. I think that it needs to be supported in terms of time and money’. P10
Accreditation competency requirements:	‘More [competencies] honestly’. P2
Competencies for systems thinking, sustainable food systems, public health	‘Some kind of competency just to make sure that there's some exposure to [systems thinking]’. P6
	‘Increased professional competencies around systems thinking and public health. We have the [Standards of Professional Performance] in Public Health and Community Nutrition…but I'm wondering, you know, if they get lost. It's such a giant document’. P11
	‘I do think having more water‐based competencies would help us think about systems. Water is this ultimate connector. And we don't even talk about water in our [program]. And that could certainly be a way that we talk about systems’. P13
Awareness of the value of systems thinking	‘I think if [other educators] saw the results and the outcomes from whatever topic they're involved with. If they could see the confidence it brings to their students. Even if it's a challenging topic’. P9
	‘I do find that just awareness, like being aware and asking the right questions is sometimes very helpful if you don't have the knowledge, at least understanding that you don't know it’. P6
	‘One thing that I think is helpful is showing that job seekers are looking for people that have systems thinking’. P1
**Dietetics Profession**	
Integration in education and practice	‘I think in the next iteration of our standards, we need to explicitly say the value of food systems work, perhaps relying on some of the work that has been done within this space and just making sure that it is in our curriculum across all fields. We need to find preceptors that will help support [systems thinking] and then [accredited program] directors are intentionally integrating this into our work. On the other end, we have it as part of our [professional development] portfolio and more explicitly showcase it as a valued part of continuing education’. P2
Continuing education	‘Ideally, to have a systems thinking course or a CEU or a resource that's aligned with systems thinking would be lovely to have’. P6
Professional Guidance	‘…when there was a President's call from the [U.S.] White House [nutrition and food security, 2022], everybody was focused on “this is what we need right now.” We weren't focused on systems, but that's what we need for food security. What are the bigger policy and long‐term strategies? In the [professional organization] world, there are stakeholders that have more influence…it doesn't help us with the kind of systems thinking that we need to do because then we're focused on economics all the time…short term vs long term’. P3
Culture	
Internal	‘Our historical way of teaching, that reductive way of teaching…I feel like I'm pushing against those norms in the field in considering the bigger picture and thinking how different things impact people's lives’. P11
	‘I think the main challenge is helping to… I don't want to say change the students worldview, but it almost feels like that. Because your undergraduate education, you're so trained, at least in nutrition and dietetics, like, this is the fact ‐ now spit it back out to me. Memorize this, which is important. We need to memorize all the macronutrients, the vitamin needs, and the DRI. And we need to memorize the way atherosclerosis develops in the body, like all of that is important to memorize. And then we're also then challenging the nutrition and dietetics professional to go beyond critical thinking to thinking about systems. And we just don't have, quite honestly, probably the skill set at academic institutions’. P1
External	‘When the heads of Agricultural Research Station were talking to us, they said “You know, we have a lot of nutrition positions, but we don't hire a nutritionist. We don't hire dietitians. By the way, what is a dietitian?” I had to explain it to this person and help them spell it properly. Didn't even know what a dietitian was. And it's such a threat to our field’. P2

### 3.1 Individual

Educators described individual characteristics contributing to their systems thinking integration from categories such as personal attributes; multi‐, inter‐ and trans‐disciplinary experiences; perspective seeking; relationship building; or resistance to change. Personal attributes reported as facilitators to systems thinking were identified from codes that included a growth mindset, values‐based education (curriculum underpinned with positive human values) [66], and risk‐taking. A growth mindset was described by one educator as ‘a desire to do better and to make a greater impact’ (P11). Another educator highlighted values‐based education, ‘figuring out what you want or what you're passionate about and having the confidence to make a change’ (P5). One participant described a risk‐taking approach.

*Our [program] was going to be doing food systems…nobody else was doing that…and there was no one to talk to about it, really. I know about as much as anybody else at that point anyway, you know? So, let's do it.* (P12)

Educators described experiences working across and within food systems to empower systems thinking, including working with producers, grocers and suppliers, distributors, consumers and managing the waste stream. One participant stated that perspective‐seeking and building relationships facilitated systems thinking, ‘just getting together with like‐minded individuals and having a conversation, sometimes it's that simple’ (P10). Alternatively, resistance to change was described as a barrier.

*I think for some people, it feels personal, you know, making shifts to dietetic education suggests that perhaps your way of thinking is outdated.* (P11)

### 3.2 Nutrition and Dietetics Education

Educators described how nutrition and dietetics education could facilitate or act as a barrier to teaching systems thinking. This included educator training, the availability of resources, accreditation competency requirements and an awareness of the value of systems thinking.

Educators described multi‐, inter‐ and transdisciplinary training to promote teaching systems thinking.

*My clinical training [at a children's hospital] was amazing. And that was very interdisciplinary, but also really great because I got to work in the community.* (P10)

*…when I earned my Master's in Public Health…that upstream approach and thinking about all of these health challenges. I think it has pushed me to realize the upstream approach is thinking about systems.* (P1)

Pedagogy and andragogy training pursued as educators were noted to facilitate systems thinking. In addition, sustainable food systems training was also described as a catalyst, as one educator explained, ‘I was fortunate with my doctorate to be able to take classes in the school of sustainability’ (P2).

Barriers to integrating systems thinking in education included a lack of systems thinking training in dietetics.

*I don't think a lot of people have had exposure to [systems thinking]. They might do some systems thinking in some way ‐ but they've never had it put in a framework.* (P6)

Limited educator skillsets were represented by statements from educators when describing two categories with overlapping traits: difficulty teaching how to navigate complexity and a limited knowledge base in sustainable food systems. Participants reported challenges in teaching complexity and in addressing sustainable food systems.

*There is this reductionist way of teaching and assessing learning objectives that emphasizes the black and white, not the gray. Some of the challenge of systems thinking, often there's not one right answer, and so it's not as easy to grade. It's…more applied work.* (P11)

*It's hard. I ask students, “How many of you actually have an eating pattern that you follow and know that you're meeting the dietary guidelines?” [Students] think it's too hard… and we're not even talking about sustainability yet.* (P3)

Resources, which included curricular resources, time, access to funding and mentorship/support, were requested by educators. Access to curricular resources was mentioned frequently, educators desired examples, case studies, example modules or simulated activities that could be integrated. Educators noted a lack of ready‐to‐use materials.

*If you were to ask me to take system thinking and incorporate it into an activity that's part of core dietetics training, I would probably figure something out, but it would probably take quite a while to figure it out.* (P4)

While educators reported time to devote to curricular building as a facilitator, educators also described a ‘perceived lack of time’ as a barrier. This included limited time for deep engagement in new topics and a lack of time for building external partners and relations.

*I felt like I never had time to do anything. I might have these great ideas, but I certainly never had the time to do the work involved in making it a really useful product.* (P12)

Most educators reported that accreditation competency requirements specific to systems thinking would drive integration in nutrition and education.

*I think dietitians would have a greater impact if we were trained more in systems thinking. Critical thinking is included in our competencies. We're really, we're missing the mark. Why not also focus on systems thinking [competencies]?* (P1)

Participants spoke positively about increasing awareness of the value of systems thinking but noted the lack of educational and credentialing incentives. They described missed opportunities:

*If it's not showing up in the competencies and standards, and it's not showing up on the registration exams, there's no real motivation or penalties or consequences if people don't include it. Adding a little bit of motivation by requiring it, but then also, you have to follow that up with enough tools that have been developed that people can pull off the shelf so that all the busy people can do it. Because I'd like to think that a lot of people are excited about system thinking and would implement it if there were tools that were easy for them to swap out.* (P4)

*I think people need to probably recognize that they're already using a lot of the tenets of systems thinking in their approach. I think they might not think of it as systems approach.* (P5)

However, there was a divergent view from one participant who expressed scepticism about mandated education, suggesting standards might not inspire genuine interest or improvement in education quality.

### 3.3 Dietetics Profession

Educators highlighted the dietetics profession's role when describing systems thinking integration in education and practice, continuing education, professional guidance and the profession's culture. They indicated systems thinking could be taught in nutrition and dietetics education and then integrated into practice through new practitioners entering the workplace, preceptor training and continuing education.

*I think it has work to come out in the education model and then into practice. Because even now, when we're teaching, it only takes one instructor to change the educational model at a place. Whereas it's a way bigger lift to get preceptors to change what they're doing.* (P13)

Educators emphasized the need for practice resources and continuing education that supports teaching systems thinking.

*[We need] example case studies, example resources, probably professional development for dietitians, [continuing professional education] credits, focused on integrating systems thinking and education at all levels. In that 200‐level class that all undergrad students have to take in nutrition that all of our departments are offering for that Gen Ed, all the way up to the master's degree.* (P1)

Educators also reported guidance from professional organizations would facilitate adoption, such as a statement recognizing the importance of systems thinking in navigating complexity.

*I think it would be appropriate for the [professional organizations] to put out resources and insights… system thinking is important for dietetics no matter what area of the profession you end up in, and here are some trainings around what it is.* (P7)

The profession's culture emerged as a barrier to systems thinking integration. Educators described an internal challenge, a traditional or reductionist approach from a curricular standpoint.

*Perhaps a tendency to keep us pegged to traditional areas like food service, for example…probably unpinning it to areas where people may think there's no relationship – there might be some challenges.* (P9)

*I just try to get it on their radar screen period… our program is so clinically focused and has been for so many years. I'm…that public health nutrition person, like I embody that, and so I just try to get them to think beyond clinical MNT…like the social determinants of health…the political determinants of health…we try to talk about advocacy…right now, that's not part of the curriculum.* (P10)

Educators also noted the profession of nutrition and dietetics is poorly recognized or represented in sustainability or food systems spaces outside of dietetics.

## 4 Discussion

This qualitative study provided insight into educators' perspectives on incorporating systems thinking into dietetics education. The interconnectedness among the themes of individual characteristics, nutrition and dietetics education, and the dietetics profession highlights how changes at any level can influence the entire system.

Building on these insights, research indicates that effective systems thinking integration, particularly in health sciences education, must start with faculty development [24]. Educators in this study reported a lack of food systems knowledge, training and experience as obstacles to systems thinking integration. This is supported by previous literature that reveals sustainable food systems training [67, 68, 69, 70, 71, 72] and multi‐, inter‐ and transdisciplinary training as areas for growth in the nutrition and dietetics profession [72, 73, 74]. Having concrete examples of how to apply systems thinking in nutrition and dietetics was highly desired, which is supported by research on systems thinking adoption in other fields [75]. In addition to developing new activities, there was a demand for competencies specific to systems thinking. This is echoed by recent research that reports curricular reform is an area for improvement if dietetics graduates are to remain relevant in an ever‐evolving practice environment [76]. A perceived lack of time, which was reported in this study, has also been described as a challenge when implementing new material within the dietetics curriculum, whether it be nutrition‐focused physical exam [73, 77]; activities related to sustainable, resilient and healthy food and water systems [78]; or precepting interns [79].

Individual qualities such as a desire for life‐long learning, taking risks, systems thinking, developing collaborative relationships and seeking diverse perspectives have been documented as essential for navigating future practice in the dietetics profession [80]. Facilitating individual qualities could influence educators' ability to integrate systems thinking into their teaching, which impacts the broader dietetics profession by shaping the practices of future dietitians.

In the US, entry‐level dietetics practice requirements and the credentialing examination are developed by surveying recent graduates. In the most recent US practice audit, more than 30% of respondents worked in clinical positions, while 40% were reported to be in ‘other’ positions that were not described [81]. Emerging practice areas might not be well captured in practice audits, leaving new areas of practice information of the US registered dietitian credentialing exam. Nutrition and dietetics education is geared towards preparing future professionals for entry‐level practice and also meeting accreditation competencies. Educators in this study reported an educational focus on clinical dietetics, this result, combined with a potential gap in capturing emerging practice areas on the credentialing exam, could lead to a systemic lack of innovation in the profession. An Australian study of graduate students revealed a similar finding, with students reporting a desire for additional support in ‘diverse areas of practice’ to feel more prepared for practice [82].

Educators described the concurrent incorporation of systems thinking into education and practice as essential for successful integration; literature in higher education also advocates for integrating systems thinking rather than treating it as a standalone concept [40]. Integration into curricula, coupled with an emphasis on values‐based education and metacognition, aligns with calls for more holistic and applied learning approaches in higher education, particularly in sustainability [83], sustainable development [84, 85] and health‐related fields [86]. This study found that transdisciplinary training, specifically in public health, facilitates systems thinking. This finding is consistent with previous research indicating that systems thinking in public health nutrition enables adaptation to complex environments [87, 88]. Additionally, education and practice resources and continuing education have also been found to facilitate the adoption of sustainable food systems [89], which educators in this study have highlighted as key to teaching systems thinking.

A reductionist approach within dietetics culture may explain why educators identified obstacles such as a lack of understanding of the dietitian's role and value among external partners and in diverse practice settings [90]. Previous literature has noted that a lack of external partnerships and collaboration are barriers to improved outcomes in the nutrition profession [91] and has highlighted the need to raise awareness about dietitian's roles [92]. Furthermore, nutrition and dietetics educators desired guidance from influential professional groups to elevate the importance of systems thinking in education and practice. Similarly, the nursing profession states that guidance from professional organizations is an important advocacy route for the profession [93].

### 4.1 Limitations

This study used both purposive and theoretical sampling to gather detailed data from a particular group. Although this method is effective for deep insights from a target population [63], it restricts how broadly the results can be applied to other groups. While the participant group was well represented regarding US geography and accredited program type, it does not represent educator experiences from countries outside of the US. Most participants identified only as White. Consequently, the lack of diversity in the sample might mean the study does not fully capture dietetics educators' viewpoints and experiences from different racial and ethnic backgrounds. The participant group was mainly women, reflecting the gender makeup in dietetics, but this also limits the study. Additionally, the primary researcher's position as a woman, RDN and nutrition and dietetics educator who has invested time into developing sustainable food systems content in the profession may have influenced the interpretation of the findings.

## 5 Conclusions

Advancing nutrition and dietetics education so future professionals can navigate complex systems requires a multifaceted approach. This study highlighted the interconnectedness between individual, educational and professional levels, underscoring the importance of a systemic approach to prepare future dietitians for complex challenges.

Characteristics such as a growth mindset, values‐based approach to learning and practicing, risk‐taking, exploration of diverse perspectives, and relationship‐building should be integrated into pedagogical and andragogical strategies. Engaging with new and emerging practice areas, in addition to a clinical focus, could facilitate a systems‐oriented view of nutrition that includes sustainable food systems and public health. Simultaneously creating and evaluating faculty and practitioner development programs could enhance systems thinking and overcome commonly cited barriers such as resistance to change, and a lack of training and resources. Concurrently revising accreditation, curricular, entry‐level practice requirements and the registered dietitian credentialing exam could foster the inclusion of systems thinking, which could create systemic change. Investigating entry‐level practice requirements in emerging and innovative areas might help promote the profession's growth. Accrediting bodies and professional‐facing organizations can advocate for a shift toward systems thinking by providing guidance, resources and professional development opportunities. Finally, embracing a cultural shift towards systems thinking and collaborating across disciplines might enhance the profession's ability to meet visionary goals and address complex food and nutrition challenges.

## Author Contributions

**Erin Bergquist:** conceptualization (lead), writing – original draft (lead), funding acquisition (lead), formal analysis (lead), writing – review and editing (equal). **Lyndi Buckingham‐Schutt:** formal analysis (supporting), review and editing (equal). **Scott Smalley:** formal analysis (supporting), review and editing (equal). **Christina Campbell:** writing – review and editing (equal). **Awoke Dollisso:** writing – review and editing (equal). **Shuyang Qu** ‐ writing – review and editing (equal). All authors agree with the manuscript. The content of this manuscript has not been published elsewhere.

## Ethics Statement

The Institutional Review Board, in the Office of Research Ethics at Iowa State University, reviewed and granted exemption on September 8, 2023, under federal regulation (IRB ID 23‐257): 2018‐2 (ii): Research that only includes interactions involving educational tests (cognitive, diagnostic, aptitude, achievement), survey procedures, interview procedures or observation of public behaviour (including visual or auditory recording) when any disclosure of the human subjects' responses outside the research would not reasonably place the subjects at risk of criminal or civil liability or be damaging to the subjects' financial standing, employability, educational advancement or reputation.

## Conflicts of Interest

The authors declare no conflicts of interest.

### Peer Review

The peer review history for this article is available at https://www.webofscience.com/api/gateway/wos/peer-review/10.1111/jhn.70036.

## Transparency Declaration

The lead author affirms that this manuscript is an honest, accurate and transparent account of the study being reported. The reporting of this work is compliant with quantitative research design. The lead author affirms that no important aspects of the study have been omitted and that any discrepancies from the study have been explained.

## Data Availability

Research data are not shared.
